# Effect of CYP3A4*22 and *PPAR-α* Genetic Variants on Platelet Reactivity in Patients Treated with Clopidogrel and Lipid-Lowering Drugs Undergoing Elective Percutaneous Coronary Intervention

**DOI:** 10.3390/genes11091068

**Published:** 2020-09-11

**Authors:** Thomas O. Bergmeijer, Alfi Yasmina, Gerrit J. A. Vos, Paul W. A. Janssen, Christian M. Hackeng, Johannes C. Kelder, Shefali S. Verma, Marylyn D. Ritchie, Li Gong, Teri E. Klein, ICPC investigators, Anthonius de Boer, Olaf H. Klungel, Jurriën M. ten Berg, Vera H. M. Deneer

**Affiliations:** 1 Department of Cardiology, St. Antonius Hospital, 3435 CM Nieuwegein, The Netherlands; t.bergmeijer@antoniusziekenhuis.nl (T.O.B.); g.vos@antoniusziekenhuis.nl (G.J.A.V.); p.janssen@antoniusziekenhuis.nl (P.W.A.J.); keld01@antoniusziekenhuis.nl (J.C.K.); jurtenberg@gmail.com (J.M.t.B.); 2 Division of Pharmacoepidemiology and Clinical Pharmacology, Utrecht Institute for Pharmaceutical Sciences, Utrecht University, 3584 CG Utrecht, The Netherlands; seribumelati@gmail.com (A.Y.); a.deboer@uu.nl (A.d.B.); O.H.Klungel@uu.nl (O.H.K.); 3 Department of Pharmacology, Faculty of Medicine, Lambung Mangkurat University, Banjarmasin 70114, Indonesia; 4 Department of Clinical Chemistry, St. Antonius Hospital, 3435 CM Nieuwegein, The Netherlands; c.hackeng@antoniusziekenhuis.nl; 5 Department of Genetics, Center for Translational Bioinformatics, Institute for Biomedical Informatics, University of Pennsylvania, Philadelphia, PA 19104, USA; shefali.setiaverma@pennmedicine.upenn.edu; 6 Department of Biomedical Data Science, Stanford University, Stanford, CA 94305, USA; marylyn@pennmedicine.upenn.edu; 7 Department of Biomedical Data Science and Department of Medicine, Stanford University, Stanford, CA 94305, USA; lgong@stanford.edu (L.G.); tklein@stanford.edu (T.E.K.); 8 Department of Clinical Pharmacy, Division of Laboratories, Pharmacy, and Biomedical Genetics, University Medical Center Utrecht, 3584 CG Utrecht, The Netherlands

**Keywords:** clopidogrel, PPAR, statin, fibrate, CYP3A4, genotyping, pharmacogenomics, PCI, personalized medicine

## Abstract

This study aims to determine whether genetic variants that influence *CYP3A4* expression are associated with platelet reactivity in clopidogrel-treated patients undergoing elective percutaneous coronary intervention (PCI), and to evaluate the influence of statin/fibrate co-medication on these associations. A study cohort was used containing 1124 consecutive elective PCI patients in whom CYP3A4*22 and *PPAR-α* (G209A and A208G) SNPs were genotyped and the VerifyNow P2Y_12_ platelet reactivity test was performed. Minor allele frequencies were 0.4% for CYP3A4*22/*22, 6.8% for *PPAR-α* G209A AA, and 7.0% for *PPAR-α* A208G GG. CYP3A4*22 was not associated with platelet reactivity. The *PPAR-α* genetic variants were significantly associated with platelet reactivity (G209A AA: −24.6 PRU [−44.7, −4.6], *p* = 0.016; A208G GG: −24.6 PRU [−44.3, −4.8], *p* = 0.015). Validation of these *PPAR-α* results in two external cohorts, containing 716 and 882 patients, respectively, showed the same direction of effect, although not statistically significant. Subsequently, meta-analysis of all three cohorts showed statistical significance of both variants in statin/fibrate users (*p* = 0.04 for *PPAR-a* G209A and *p* = 0.03 for A208G), with no difference in statin/fibrate non-users. In conclusion, *PPAR-α* G209A and A208G were associated with lower platelet reactivity in patients undergoing elective PCI who were treated with clopidogrel and statin/fibrate co-medication. Further research is necessary to confirm these findings.

## 1. Introduction

Dual antiplatelet therapy (DAPT), i.e., the combination of clopidogrel and aspirin, significantly reduces the risk of cardiovascular death, myocardial infarction, or urgent target vessel revascularization, compared to aspirin monotherapy in patients undergoing percutaneous coronary intervention (PCI) [1]. However, the response to clopidogrel varies between patients. This variability is caused by several factors, including polymorphisms in genes encoding for enzymes involved in the metabolism of clopidogrel, drug interactions, a history of diabetes, smoking status, body mass index (BMI), and age [2,3,4,5,6,7,8,9].

Clopidogrel is a prodrug activated by cytochrome P450 enzymes through two oxidative steps into an active metabolite that inhibits ADP-induced platelet aggregation. CYP2C19 contributes to both steps and CYP3A4 contributes to the second step [10]. Factors affecting CYP2C19 and CYP3A4 expression and activity may influence the blood concentration of clopidogrel’s active metabolite, and eventually platelet aggregation. CYP2C19*2 and *3 have been shown to affect platelet reactivity and cardiovascular outcome in patients treated with clopidogrel, while the data about the clinical relevance of the CYP2C19*17 gain-of-function allele is conflicting [11,12,13]. A GWAS study in a large Amish population indicated that approximately 70% of the variability in clopidogrel response may be due to genetic factors, while CYP2C19*2 being responsible for approximately 12% of the overall variation in platelet reactivity [14]. This suggests that other genetic factors influencing clopidogrel efficacy remain to be identified. 

The CYP3A4*22 genetic variant has been shown to decrease the expression of CYP3A4 [15]. Although the minor allele frequency in the European population is only about 5%, it may serve as a marker to predict the response to drugs metabolized by CYP3A4 [15,16]. Two linked peroxisome proliferator-activated receptor alpha (*PPAR-α*) genetic variants (G209A and A208G) have also been identified as genetic determinants that affect CYP3A4 expression, explaining ~5% and ~9% of the variation in CYP3A4 protein and activity level, respectively, which might influence clopidogrel active metabolite level and thereby platelet reactivity [17]. With a minor allele frequency of 27% and 28%, respectively, in the European population, those SNPs are more common [15]. Statins and fibrates are both ligands of PPAR-α, and the resulting activation of PPAR-α will therefore reduce platelet aggregation [18]. Furthermore, CYP3A4*22 and the two variants of the *PPAR*-*α* gene were associated with a lower required dose of CYP3A4-metabolized drugs such as simvastatin and atorvastatin [16,17]. 

So far, the available data describing the influence of *CYP3A4* genetic variations on platelet reactivity in patients treated with clopidogrel is limited, and as far as the authors are aware the influence of *PPAR-α* on platelet reactivity in the presence of statin co-medication has not been studied.

Since platelet reactivity in clopidogrel-treated patients who underwent PCI has been associated with cardiovascular outcomes [19,20], the influence of genetic factors and co-medications on this on-treatment platelet reactivity is relevant for clinical practice. In this study, we aimed to investigate the association between the genetic variations of the *CYP3A4* and *PPAR-α* genes and platelet reactivity in clopidogrel-treated patients undergoing elective PCI, and to evaluate the influence of statin/fibrate co-medication on these associations.

## 2. Materials and Methods 

### 2.1. Study Population

The study population included all consecutive patients who underwent non-urgent PCI with stent implantation, in whom genotyping for the CYP2C19*2 and *3 alleles was performed between July 2010 and May 2013 in the St. Antonius Hospital, Nieuwegein, The Netherlands. *CYP2C19* genotyping, and subsequent adjustment of antiplatelet therapy based on a combination of platelet function testing, genotyping results and clinical risk factors, was performed as part of routine patient care in those patients [21]. According to hospital protocols, the remaining blood from the genotyping samples could be used for further research, in an anonymized manner, if the patient did not object. For the current analysis, CYP3A4*22 and *PPAR-α* genotyping was performed. 

Patients were excluded from the analysis if they had a history of stroke or transient ischemic attack, were treated with oral anticoagulants, had a platelet count below 100 × 10^9^/L, or received a glycoprotein IIb/IIIa-inhibitor before a blood sample was collected. All patients were adequately pre-treated with clopidogrel (defined as 75 mg daily for ≥5 days, a loading dose of 300 mg ≥ 6 h or 600 mg ≥ 2 h before testing) and aspirin (80–100 mg daily). 

The study complies with the Declaration of Helsinki and received approval from the hospital’s medical research ethics committee (Verenigde Commissies Mensgebonden Onderzoek/VCMO). Approval for this registry included a waiver of informed consent.

### 2.2. Exposure and Outcome

The exposures in this study were the genetic variations of *CYP3A4* (CYP3A4*22 or rs35599367) and *PPAR-α* (G209A or rs4253728, and A208G or rs4823613) genes. Blood samples for DNA analysis were obtained with K3-EDTA tubes as part of routine patient care for *CYP2C19* genotyping. Coded blood samples and DNA isolates were stored at −70 °C. The samples were genotyped for CYP3A4*22, *PPAR-α* G209A, *PPAR-α* A208G, and CYP2C19*2 and *3 using the StepOnePlus^®^ Real-Time PCR system. The TaqMan^®^ SNP Genotyping Assay, which includes two allele-specific probes and PCR primer pairs to detect specific genetic polymorphism targets, was used. The StepOnePlus^®^ software was used to determine the genotype of individual patients. The potential effect modifier in this study was statins/fibrates co-medication. 

The outcome was on-treatment platelet reactivity, defined as the absolute level of platelet reactivity during treatment with clopidogrel. Platelet reactivity was measured with the VerifyNow^®^ P2Y_12_-assay (Werfen, Barcelona, Spain). All platelet reactivity measurements were performed within 2 h after blood sample collection. Platelet reactivity was expressed in P2Y_12_ reaction unit (PRU). High on-clopidogrel platelet reactivity (HPR) was defined as the platelet reactivity of ≥236 PRU [19], but the same analysis was performed for the >208 PRU cut off value [22].

Several potential confounding variables were a priori considered based on previous publications, namely age, sex, current smoking, body mass index (BMI), diabetes, prior myocardial infarction, impaired renal function, clopidogrel loading before PCI (as compared to patients who were already using a maintenance dose), co-medication (proton pump inhibitors, statin/fibrate use, calcium channel blockers), and CYP2C19 metabolizer status. Impaired renal function was defined as serum creatinine level ≥ 200 µmol/L. The CYP2C19 metabolizer status was categorized into three groups: normal metabolizer (*1/*1 genotype), intermediate metabolizer (*1/*2 or *1/*3 genotype), and poor metabolizer (*2/*2, *3/*3, or *2/*3 genotype). Information on these potential confounders was obtained from hospital records.

### 2.3. Validation Cohorts

To validate our findings regarding the *PPAR-α* G209A and *PPAR-α* A208G SNPs, two different external databases were used. The first validation cohort consisted of the patients described in the POPular study. This observational single center study analyzed the predictive value of different platelet function tests on clinical outcome in patients using clopidogrel and was performed in the same hospital as our current analysis [19]. All patients undergoing elective PCI in whom platelet function testing was performed using the VerifyNow P2Y_12_ assay and from whom a DNA sample was available for the genotyping of both PPAR SNPs were selected. The technique used for the genotyping was the same as described for the main analysis.

The second validation cohort consisted of all patients in the genome wide association study (GWAS) subgroup of the International Clopidogrel Pharmacogenomics Consortium (ICPC) database. The aim of the ICPC is to find novel genetic markers which influence clopidogrel efficacy using GWAS and candidate gene approaches, combined with pharmacodynamic and clinical outcome data [23,24]. All clopidogrel-treated patients undergoing elective PCI were selected in whom ADP-stimulated platelet function testing was performed. Because different platelet function tests were used among different cohorts, a standardized ADP-induced platelet reactivity measure was used (*z*-score). Therefore, HPR status could not be determined in this validation cohort. Calculation of the standardized antiplatelet measure has been described in the ICPC design paper [23]. For GWAS the Illumina Omni Express with Exome chip was used. The PPAR SNPs were available in all patients as part of the GWAS analysis. Due to missing baseline variables, no multivariate analysis was performed in the ICPC cohort analysis.

### 2.4. Statistical Analysis

Continuous variables are presented as mean ± SD, and categorical variables are presented as proportions. The on-treatment platelet reactivity was normally distributed. Chi-square tables were used to compare the observed number of each genotype with the expected number for a population in Hardy–Weinberg equilibrium (*p* > 0.05). The pairwise linkage disequilibrium between *PPAR-α* G209A and *PPAR-α* A208G was calculated. Multivariate linear regression analysis was used to test the association between the genetic variants and on-treatment platelet reactivity as continuous variable, adjusted for confounders. Multivariate logistic regression analysis was conducted to test the association between the genetic variants and HPR, adjusted for confounders. Stratified analyses were conducted for patients with statin/fibrate co-medication users versus non-users. A recessive model was used in all the analyses. To compare the main analysis to the validation cohorts, a meta-analysis was performed with an inverse variance method, using a random effects model. Statistical analyses were conducted with SPSS 24 and R 3.1.3.

## 3. Results

### 3.1. Study Cohort

This study included 1124 patients who underwent elective PCI. Most of the patients were male (75.4%), mean 63.9 years of age (±10.8 years), with some overweight (BMI 27.5 ± 4.2 kg/m^2^), and with a history of hypertension (83.3%). At the time of PCI, 88.6% of patients were using statin and/or fibrate therapy, mostly simvastatin (67.7%) or atorvastatin (19.4%), while 3 patients (0.3%) were using a fibrate. Of all patients, 0.4% were homozygous carriers of the CYP3A4*22 allele, 6.9% were homozygous for the *PPAR-α* G209A minor allele, and 7.0% were homozygous for the *PPAR-α* A208G minor allele. Poor CYP2C19 metabolizer status was present in 2.6% of patients (Table 1). All genetic variants were in Hardy–Weinberg equilibrium (*p* > 0.05). Strong linkage disequilibrium was observed between *PPAR-α* G209A and *PPAR-α* A208G (*r*^2^ = 0.96).

**Figure 1:**
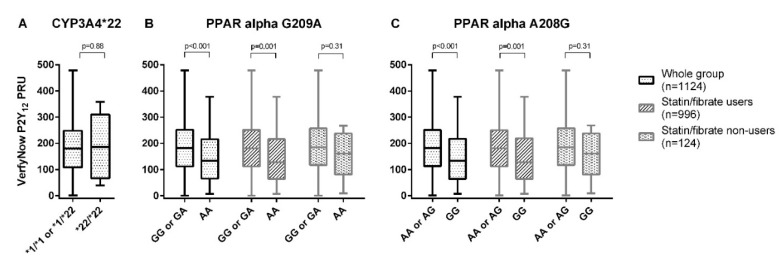
The on-treatment platelet reactivity, as measured with the VerifyNow^®^ P2Y_12_ assay for CYP3A4*22 (**A**), *PPAR-α* G209A (**B**), and *PPAR-α* A208G (**C**).

There was no difference in the on-treatment platelet reactivity between the homozygous CYP3A4*22 allele carriers versus the heterozygous or non-carriers (*1/*22 or *1/*1) (*p* = 0.88) (Figure 1a).

A significantly lower on-treatment platelet reactivity was found in those with the *PPAR-α* G209A AA genotype compared to the GG or GA genotype (non-adjusted mean difference −40.1 PRU [95%CI −62.1, −18.0], *p* < 0.001) (Figure 1b, Table 2). 

A significant difference was also found between the patients with the *PPAR-α* A208G GG genotype and the patients with the AA or AG genotypes (non-adjusted mean difference = −39.1 PRU [95%CI −60.9, −17.3], *p* < 0.001) (Figure 1c, Table 2). After adjustment for possible confounders, the effect for both PPAR SNPs was still significant (Table 2). The subgroup analysis for statin/fibrate co-medication showed a comparable effect for both the statin/fibrate users and non-users, although not statistically significant in the subgroup of statin/fibrate non-users, due to the lower number of patients in each subgroup (Table 2). Results for *PPAR-α* G209A analyzed in an additive or dominant model are shown in Online Supplemental Figure S1, while the results for each individual statin are shown in Online Supplemental Figure S2.

A lower incidence of HPR > 208 PRU was observed in those with the *PPAR-α* G209A AA genotype compared to the heterozygous and non-carriers of the minor allele (28.6 vs. 39.5%, Odds Ratio 0.61 [95%CI 0.37, 1.02], *p* = 0.06) and in those with *PPAR-α* A208G GG genotype (29.1 vs. 39.5%, OR 0.63 [95%CI 0.38, 1.04], *p* = 0.07) (Table 3). 

A subgroup analysis for the association with HPR in statin/fibrate users and non-users showed a similar trend in results as with the continuous platelet reactivity outcome. However, the associations were not significant, both for univariate and multivariate analysis. Results for the HPR ≥ 236 PRU cut off value are presented in Supplemental Table S1.

### 3.2. Validation Cohorts

**Figure 2:**
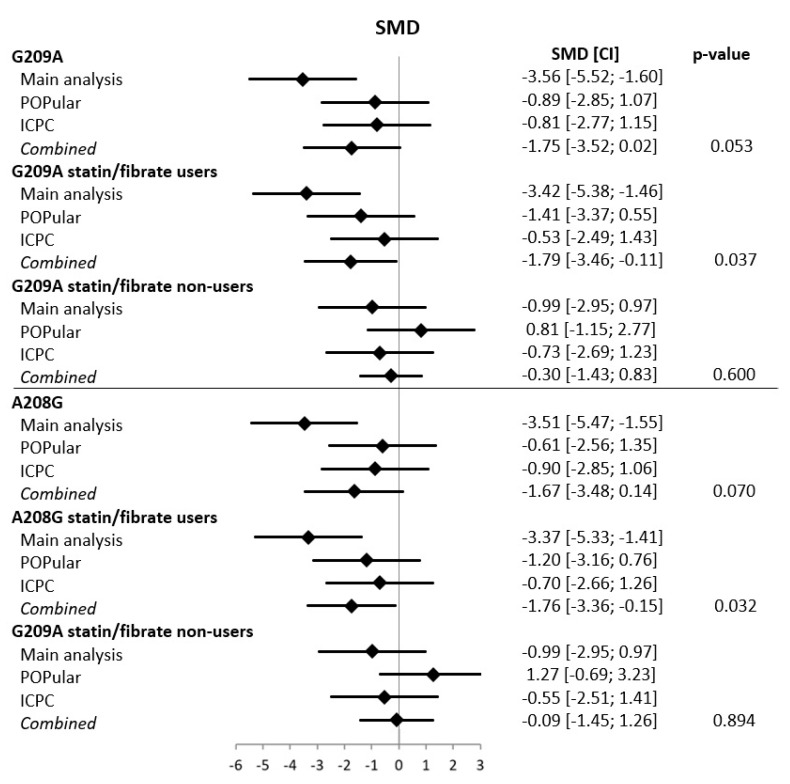
Meta-analysis for G209A and A208G variants in the main analysis and the POPular and ICPC validation cohorts. CI = confidence interval, SMD = standardized mean difference. For the meta-analysis, a random effects model was used.

Baseline characteristics of the POPular validation cohort (*n* = 729) and the ICPC validation cohort (*n* = 882) are shown in Supplemental Table S2. In general, clinical characteristics and gene frequencies were comparable between the three cohorts. The lower platelet reactivity found in the main analysis for the *PPAR-α* G209A AA and the *PPAR-α* A208G GG genotype was also found in the POPular validation cohort, but the effect was less pronounced and not statistically significant (G209A AA: −10.5 PRU [95%CI −33.8, 12.7], *p* = 0.35; A208G GG: −7.0 [95%CI −29.5, 15.6], *p* = 0.51) (Supplemental Figure S3 and Table S3). Still, the odds ratio for HPR > 208 PRU was comparable to the results found in the main analysis (G209A AA: OR 0.67 [95%CI 0.36; 1.23], *p* = 0.22; A208G GG: OR 0.75 [95%CI 0.41, 1.34], *p* = 0.37) (Supplemental Table S4). When stratified to statin/fibrate users versus non-users there was a lower platelet reactivity in statin/fibrate users (G209A AA: −18.1 PRU [95%CI −44.6, 8.5], *p* = 0.18; A208G GG: −14.5 [95%CI −39.8, 10.7], *p* = 0.18), but a higher platelet reactivity in the statin/fibrate non-users (G209A AA: +13.6 [95%CI −33.7, 60.9], *p* = 0.57; A208G GG: +24.6 [95%CI −25.0, 74.2], *p* = 0.33) (Supplemental Figure S3 and Table S3). In the ICPC validation cohort both *PPAR-α* SNPs were also associated with numerically lower ADP-induced platelet reactivity, but without statistically significant differences (Supplemental Table S5). This held true for both the whole group and for the statin/fibrate user or non-user subgroups. When all three databases where combined using a random effects meta-analysis model, platelet reactivity was significantly lower for both the *PPAR-α* G209A and A208G homozygous minor allele carriers in the subgroup of statin users (*p* = 0.04 for G209A and *p* = 0.03 for A208G) (Figure 2), although the effect is driven by the results of the main analysis. There was no significant effect found in the combined analysis for the subgroup of statin/fibrate non-users.

## 4. Discussion

In this analysis, clopidogrel-treated patients undergoing elective PCI who were homozygous for the *PPAR-α* G209A and A208G minor allele had a significantly lower platelet reactivity when measured with the VerifyNow P2Y_12_ platelet function test compared to heterozygous or wild-type patients. In two external validation cohorts, we also found a lower platelet reactivity associated with those SNPs, although this difference was not statistically significant. The effect seems to be driven by the patients using statin or fibrate co-medication. CYP3A4*22 was not found to be associated with a difference in platelet reactivity, but this analysis is hampered by a very low number of patients homozygous for the minor allele. 

A previous study published by Kreutz et al. did not find an association between PPAR-α and platelet reactivity [25]. This could have been due to the lower sample size, and as a consequence, the additive model that was used in their analysis. In our study, a recessive model was used, in accordance with the results of the study by Klein et al., which demonstrated that only homozygous carriers showed a decrease in PPAR-α protein and activity levels [17].

PPAR-α is one of the three PPARs found in the cell nucleus that modulate the transcription of various genes associated with lipid metabolism and inflammation. Recent studies have shown that all three types of PPAR proteins (α, β, and γ) are also expressed in the human bone marrow megakaryocytes and anucleate platelets. The proteins are susceptible to their specific endogenous and exogenous ligands, which affect platelet reactivity through a non-genomic mechanism [18,26,27]. A previous study showed that the two linked genetic variants of the *PPAR-α* gene (G209A and A208G) were associated with a reduced expression of PPAR-α, and they also directly or indirectly modulated CYP3A4, as proven by the decrease in expression and activity of CYP3A4 in the liver [17]. Based on those findings, subjects who are homozygous for the *PPAR-α* G209A or A208G minor allele are expected to have a lower expression of CYP3A4, leading to a lower blood level of the active metabolite of clopidogrel and eventually higher on-treatment platelet reactivity, compared to heterozygous or wild-type patients. 

However, there is an additional effect of statin and fibrate drugs on platelet reactivity in the other direction, leading to a net effect of lower platelet reactivity. PPAR-α has a direct effect on platelet reactivity. Ligand-activated PPAR-α will rapidly inhibit PKC-α and will suppress platelet activation and aggregation. Aside from that, ligand-activated PPAR-α increases the level of cAMP that leads to the inhibition of platelet activation [18]. It also inhibits cyclooxygenase-1, leading to inhibition of arachidonic acid-related platelet aggregation. In addition, ligand-induced PPAR-α increases the activity of nitric oxide synthase and guanylyl cyclase, leading to the inhibition of collagen-induced platelet aggregation [28]. Statins and fibrates are strong ligands for PPAR-α [29,30] and PPAR-γ [18,29]. Both types of PPARs will inhibit platelet aggregation when they are activated by statins or fibrates. Furthermore, patients who are homozygous for the *PPAR-α* G209A or A208G minor allele will have reduced expression of CYP3A4, which acts as a metabolizing enzyme for statins [17]. This results in higher blood concentration of statins and most likely fibrates. Therefore, since 88.6% of our patients were users of a statin or fibrate, the on-treatment platelet reactivity in this study was the result of the net effect of clopidogrel and statin/fibrate. In clopidogrel users who were homozygous carriers of the *PPAR-α* minor allele and were also using a statin or fibrate, the on-treatment platelet reactivity reflected a balance between the increased platelet reactivity caused by reduced expression of CYP3A4, the reduced platelet reactivity caused by the higher blood concentration of statin/fibrate and statin/fibrate-induced PPAR-α-mediated and PPAR-γ-mediated antiplatelet activity. 

CYP3A4*22 has been reported to be associated with the reduction in CYP3A4 activity [16]. This genetic variant was shown to affect the blood level or dose requirement of CYP3A4-metabolized drugs such as tacrolimus, simvastatin, and atorvastatin [16,17,30]. Our results on the effect of CYP3A4*22 on the on-treatment platelet reactivity showed a slightly increased platelet reactivity, but the association was not significant and limited by the small proportion of homozygous carriers of the minor allele in this study (*n* = 5). A previous study on the same genetic variant on the effect of clopidogrel did not include any subjects with the *22/*22 genotype, so that an additive model was used, in which no association between CYP3A4*22 and on-treatment platelet reactivity was found [25]. 

Based on previous publications, there is a strong correlation between HPR, defined as > 208 or ≥ 236 PRU, and major adverse cardiovascular events (MACE) [19,31]. In our analysis we found a non-significant correlation between both *PPAR-α* genetic variants and HPR, both for the > 208 PRU and ≥ 236 PRU cut off level (Table 3 and Supplemental Table S4). A meta-analysis by Brarr et al. showed that, on a continuous scale, every 10 unit increase in PRU was significantly associated with a 4% increased risk of the composite of death, myocardial infarction, or stent thrombosis (HR 1.04; 95% CI 1.03–1.06) [20]. In our results, clopidogrel users who were homozygous carriers of a *PPAR-α* minor allele and were statin/fibrate users had a significant 26 unit decrease in PRU (Table 2), implicating that this group of patients may have a 10% decreased risk of the composite of death, myocardial infarction, or stent thrombosis compared to the patients with heterozygous and wild-type *PPAR-α* genotypes. 

The strength of our analysis is that we can evaluate the association between the genetic variants in *PPAR-α* genes and the on-treatment platelet reactivity in a patient cohort with a large sample size, and we could validate our findings in two patient cohorts with comparable patient characteristics. We took into account the biological mechanism of ligand-activated PPAR proteins-associated platelet reactivity when evaluating the association. Since the genetic polymorphisms in *CYP2C19* have a considerable contribution in the platelet reactivity during treatment with clopidogrel, our study included the CYP2C19*2 and *3 genetic variants for the statistical analysis. Other drugs that might influence clopidogrel’s response as a result of a pharmacokinetic interaction were also included, such as the use of calcium channel blockers and proton pump inhibitors. Platelet reactivity was measured while the patients were adequately treated with clopidogrel. The VerifyNow P2Y_12_ assay, a reliable and sensitive tool to measure platelet response to clopidogrel therapy, was used to measure platelet reactivity.

This study also has its limitations. First, the frequency of CYP3A4*22/*22 was too small to draw any definite conclusions regarding its association with on-treatment platelet reactivity or HPR. Also, the subgroup of patients without statin/fibrate use was small, which results in a very wide confidence interval for the calculated odds ratios in this subgroup, and a valid multivariate analysis could not be performed. A possible difference between statin/fibrate users and non-users could have been missed. Second, our analysis was limited to elective PCI patients only, while the correlation between platelet reactivity and *CYP2C19* genotype with clinical outcome is stronger in high risk patients, for example after acute coronary syndrome (ACS) [32]. Therefore, the effect of *PPAR* SNPs on platelet reactivity and clinical outcome might also be stronger in ACS-patients. Third, multiple platelet function testing methods were used in the ICPC validation cohort, making it necessary to calculate a standardized ADP induced antiplatelet measure. This makes it difficult to compare the ICPC results to the results of the main analysis and the POPular validation cohort, in which a single platelet function test has been used in all patients. A meta-analysis approach was used to account for this difference. Finally, although both validation cohorts showed an effect on platelet reactivity for the *PPAR-α* genetic variants in the same direction as the main analysis, the effect size was smaller and the results were not statistically significant. Also, active metabolite levels were not available, but would have been useful to support our hypothesis about the mechanism of effect on platelet reactivity.

Nevertheless, the trend towards lower platelet reactivity warrant further analysis in other cohorts, focusing on patients with a higher ischemic risk and using clinical endpoint data.

## 5. Conclusions

In our analysis, linked G209A and A208G genetic variants in the *PPAR-α* gene were associated with lower platelet reactivity in elective PCI patients treated with clopidogrel and statin/fibrate co-medication. Replication in two external validation cohorts showed the same direction of effect, but without statistical significance. However, meta-analysis of the three cohorts shows that both variants are statistically significant.

## Figures and Tables

**Table 1:** Baseline characteristics.

	All Patients (*n* = 1124)
Characteristics	*n* (%)
Age, years (mean, SD)	63.9 ± 10.8
Sex (male)	848 (75.4)
BMI, kg/m^2^ (mean, SD)	27.5 ± 4.2
Smoking habit	
No	632 (56.1)
Current smoker or stopped < 6 months	247 (22.0)
Past smoker	238 (21.2)
Unknown	7 (0.6)
Disease history	
Hypertension	936 (83.3)
Diabetes	235 (20.9)
Myocardial infarction	349 (31.0)
Co-medication at baseline	
Beta-blocker	900 (80.1)
ACE inhibitor	422 (37.5)
Calcium channel blocker	314 (27.9)
ARB	204 (18.1)
Diuretic	313 (27.8)
Statin or fibrate	996 (88.6)
Simvastatin	647 (67.7)
Atorvastatin	193 (19.4)
Rosuvastatin	81 (8.1)
Pravastatin	58 (5.8)
Fluvastatin	7 (0.7)
Fibrate	3 (0.3)
Unknown/missing	9 (0.9)
Proton pump inhibitor	454 (40.4)
Impaired renal function ^‡^	9 (0.8)
Clopidogrel loading ^†^	395 (35.1)
CYP2C19 metabolizer	
Normal	821 (73.0)
Intermediate	274 (24.4)
Poor	29 (2.6)
CYP3A4*22	
MAF	6.3%
*1/*1	987 (87.8)
*1/*22	132 (11.7)
*22/*22	5 (0.4)
*PPAR-α* G209A	
MAF	25.4%
GG	630 (56.0)
GA	417 (37.1)
AA	77 (6.9)
*PPAR-α* A208G	
MAF	27.0%
AA	597 (53.1)
AG	448 (39.9)
GG	79 (7.0)

ACE = angiotensin converting enzyme, ARB = angiotensin receptor blocker, BMI = body mass index, MAF = minor allele frequency, PCI = percutaneous coronary intervention, SD = standard deviation.; ^‡^ Defined as creatinine value >200 mmol/L. ^†^, defined as clopidogrel 300 mg ≥ 6 h before testing, or clopidogrel 600 mg ≥ 2 h before testing.

**Table 2:** On-treatment platelet reactivity in carriers of recessive alleles of CYP3A4*22, *PPAR-α* G209A, and *PPAR-α* A208G.

Genetic Variant	Mean ± SD	Coefficient (95% CI)	
		Crude	Adjusted
**All patients (*n* = 1124)**			
CYP3A4*22			
*1/*1 or *1/*22 (*n* = 1119)	181 ± 96	Ref	Ref
*22/*22 (*n* = 5)	188 ± 128	6.6 (−77.6, 90.8), *p* = 0.88	NA ^‡^
*PPAR-α* G209A			
GG or GA (*n* = 1047)	184 ± 95	Ref	Ref
AA (*n* = 77)	144.0 ± 94	−40.1 (−62.1, −18.0), *p* < 0.001	−24.6 (−44.7, −4.6), *p* = 0.016 ^†^
*PPAR-α* A208G			
AA or AG (*n* = 1045)	184 ± 95	Ref	Ref
GG (*n* = 79)	145 ± 95	−39.1 (−60.9, −17.3), *p* < 0.001	−24.56 (−44.3, −4.8), *p* = 0.015 ^†^
**In statin/fibrate users (*n* = 996)**			
*PPAR-α* G209A			
GG or GA (*n* = 928)	184 ± 95	Ref	Ref
AA (*n* = 68)	143 ± 95	−40.8 (−64.2, −17.4), *p* = 0.001	−26.7 (−47.9, −5.4), *p* = 0.014 ^§^
*PPAR-α* A208G			
AA or AG (*n* = 926)	184 ± 95	Ref	Ref
GG (*n* = 70)	144 ± 96	−39.7 (−62.8, −16.6), *p* = 0.001	−26.5 (−47.5, −5.6), *p* = 0.013 ^§^
**In statin/fibrate non-users (*n* = 124)**			
*PPAR-α* G209A			
GG or GA (*n* = 115)	190 ± 100	Ref	Ref
AA (*n* = 9)	154 ± 90	−34.3 (−103.2, 32.7), *p* = 0.31	NA ^‡^
*PPAR-α* A208G			
GG or GA (*n* = 115)	190 ± 100	Ref	Ref
AA (*n* = 9)	154 ± 90	−34.3 (−103.2, 32.7), *p* = 0.31	NA ^‡^

All values are in VerifyNow PRU. CI = confidence interval, NA = not applicable, SD = standard deviation, Ref = reference. Symbols: ^†^ Adjusted for age, sex, body mass index, smoking, diabetes, previous myocardial infarction, co-medication (calcium channel blockers, statins/fibrates, proton pump inhibitors), impaired renal function, clopidogrel loading, and CYP2C19 metabolizer status. ^§^ Adjusted for all variables mentioned above, excluding statin/fibrate use. ^‡^ Due to the low number of patients, multivariate analysis was not performed in this subgroup.

**Table 3:** Odds ratio for high platelet reactivity in carriers of recessive alleles of CYP3A4*22, *PPAR-α* G209A, and *PPAR-α* A208G for the >208 PRU cut-off value.

Genetic Variant	HPR+ (*n* = 436)	HPR− (*n* = 688)	Crude OR (95% CI)	Adjusted OR (95% CI)
**All patients (*n* = 1124)**				
CYP3A4*22				
*1/*1 or *1/*22 (*n* = 1119)	434 (38.8)	685 (61.2)	Ref	Ref
*22/*22 (*n* = 5)	2 (40.0)	3 (60.0)	1.05 (0.18, 6.32)	NA ^‡^
*PPAR-α* G209A				
GG or GA (*n* = 1047)	414 (39.5)	633 (60.5)	Ref	Ref
AA (*n* = 77)	22 (28.6)	55 (71.4)	0.61 (0.37, 1.02)	0.82 (0.47, 1.43) *
*PPAR-α* A208G				
AA or AG (*n* = 1045)	413 (39.5)	632 (60.5)	Ref	Ref
GG (*n* = 79)	23 (29.1)	56 (70.9)	0.63 (0.38, 1.04)	0.83 (0.48, 1.43) *
**In statin/fibrate users (*n* = 996)**				
*PPAR-α* G209A				
GG or GA (*n* = 928)	371 (39.2)	576 (60.8)	Ref	Ref
AA (*n* = 68)	19 (27.1)	51 (72.9)	0.60 (0.35, 1.03)	0.77 (0.43, 1.38) ^§^
*PPAR-α* A208G				
AA or AG (*n* = 926)	370 (39.2)	575 (60.8)	Ref	Ref
GG (*n* = 70)	20 (27.8)	52 (72.2)	0.62 (0.36, 1.05)	0.78 (0.43, 1.38) ^§^
**In statin/fibrate non-users (*n* = 124)**				
*PPAR-α* G209A				
GG or GA (*n* = 115)	42 (43.8)	54 (56.3)	Ref	Ref
AA (*n* = 9)	3 (42.9)	4 (57.1)	0.66 (0.17, 3.04)	NA ^‡^
*PPAR-α* A208G				
GG or GA (*n* = 115)	42 (43.8)	54 (56.3)	Ref	Ref
AA (*n* = 9)	3 (42.9)	4 (57.1)	0.66 (0.17, 3.04)	NA ^‡^

CI = confidence interval, HPR = high on-treatment platelet reactivity, NA = not applicable, OR = odds ratio, Ref = reference. All *p*-values for crude and adjusted odds ratios are > 0.05. * Adjusted for age, sex, body mass index, smoking, diabetes, previous myocardial infarction, co-medication (calcium channel blockers, statins/fibrates, proton pump inhibitors), impaired renal function, clopidogrel loading, and CYP2C19 metabolizer status. ^§^ Adjusted for all variables mentioned above, excluding statin/fibrate use. ^‡^ Due to the low number of patients, multivariate analysis was not performed in this subgroup.

## Supplementary Materials

The following are available online at https://www.mdpi.com/2073-4425/11/9/1068/s1. Table S1: On-treatment platelet reactivity in carriers of recessive alleles of *CYP3A4**22, *PPAR-α* G209A, and *PPAR-α* A208G for the HPR ≥ 236 PRU cut off in the main analysis cohort. Table S2: Baseline characteristics for the main analysis cohort and both validation cohorts. Table S3: Estimates for the on-treatment platelet reactivity outcome in carriers of recessive alleles of *PPAR-α* G209A, and *PPAR-α* A208G in the POPular validation cohort. Table S4: Odds ratio for high platelet reactivity outcome in carriers of recessive alleles of *PPAR-α* G209A, and *PPAR-α* A208G in the POPular validation cohort. Table S5: Estimates for the on-treatment platelet reactivity outcome in carriers of recessive alleles of *PPAR-α* G209A, and *PPAR-α* A208G in the ICPC validation cohort. Figure S1: The on-treatment platelet reactivity in the main analysis cohort, as measured with the VerifyNow^®^ assay for PPAR alpha G209A (rs4253728), analyzed in a (a) additive model, (b) dominant model. Figure S2: The on-treatment platelet reactivity in the main analysis cohort, as measured with the VerifyNow^®^ assay for PPAR alpha G209A (rs4253728), stratified according to different statin and fibrate use. Figure S3: The on-treatment platelet reactivity, as measured with the VerifyNow^®^ assay: (a) *PPAR-α* G209A (rs4253728), (b) *PPAR-α* A208G (rs4823613) in the POPular validation cohort. Online Supplementary Data: International Clopidogrel Pharmacogenomic Consortium (ICPC) author list.
