# Supplementary material for: Effect of CYP3A4*22 and PPAR-α Genetic Variants on Platelet Reactivity in Patients Treated with Clopidogrel and Lipid-Lowering Drugs Undergoing Elective Percutaneous Coronary Intervention
Source: Genes (Basel). 2020 Sep 11;11(9):1068. doi: 10.3390/genes11091068 (PMC7564055; doi:10.3390/genes11091068)
Supplement: Supplementary file 1 [file genes-11-01068-s001.pdf]

Online Supplemental Table S1.

On-treatment platelet reactivity in carriers of recessive alleles of *CYP3A4*22, PPAR-α* G209A, and *PPAR-α* A208G for the HPR ≥ 236 PRU cut off in the main analysis cohort

Genetic variant	HPR+ (n = 324)	HPR- (n = 800)	Crude OR (95% CI)	Adjusted OR (95% CI)
**All patients (n = 1,124)**				
*CYP3A4*22*				
*1/*1 or *1/*22 (n = 1119)	322 (28.8)	797 (71.2)	Ref	Ref
*22/*22 (n = 5)	2 (40.0)	3 (60.0)	1.65 (0.27, 9.92)	3.34 (0.46, 24.27)*
PPAR-α G209A				
GG or GA (n = 1047)	308 (29.4)	739 (70.6)	Ref	Ref
AA (n = 77)	16 (20.8)	61 (79.2)	0.63 (0.36, 1.11)	0.89 (0.48, 1.67)*
PPAR-α A208G				
AA or AG (n = 1045)	307 (29.4)	738 (70.6)	Ref	Ref
GG (n = 79)	17 (21.5)	62 (78.5)	0.66 (0.38, 1.15)	0.91 (0.49, 1.68)*
**In statin/fibrate users (n = 996)**				
PPAR-α G209A				
GG or GA (n = 928)	275 (29.0)	672 (71.0)	Ref	Ref
AA (n = 68)	14 (20.0)	56 (80.0)	0.63 (0.34, 1.15)	0.86 (0.45, 1.66)§
PPAR-α A208G				
AA or AG (n = 926)	274 (29.0)	671 (71.0)	Ref	Ref
GG (n = 70)	15 (20.8)	57 (79.2)	0.66 (0.37, 1.19)	0.88 (0.46, 1.67)§
**In statin/fibrate non-users (n = 124)**				
PPAR-α G209A				
GG or GA (n = 115)	32 (33.3)	64 (66.7)	Ref	Ref
AA (n = 9)	2 (28.6)	5 (71.4)	0.63 (0.12, 3.17)	1.18 (0.08, 16.47)§
PPAR-α A208G				
GG or GA (n = 115)	32 (33.3)	64 (66.7)	Ref	Ref
AA (n = 9)	2 (28.6)	5 (71.4)	0.63 (0.12, 3.17)	1.18 (0.08, 16.47)§

Note:
*CYP3A4*22* = rs35599367, G209A = rs4253728, A208G = rs4823613
Abbreviations: CI = confidence interval, HPR = high on-treatment platelet reactivity, OR = odds ratio, Ref = reference. All p-values for crude and adjusted odds ratios are > 0.05.
* Adjusted for age, sex, body mass index, smoking, diabetes, previous myocardial infarction, co-medication (calcium channel blockers, statins/fibrates, proton pump inhibitors), impaired renal function, clopidogrel loading, and CYP2C19 metabolizer status.
§ Adjusted for all variables mentioned above, excluding statin/fibrate use

Online Supplemental Table S2. Baseline characteristics for the main analysis cohort and both validation cohorts

	Main analysis (n = 1,124)	Validation cohorts	
		POPular (n = 716)	ICPC (n = 885)
**Characteristics**	**n (%)**	**n (%)**	**n (%)**
Age, years (mean, SD)	63.9 ± 10.8	63.9 ± 10.0	64.0 ± 10.5
Sex (male)	848 (75.4)	538 (75.1)	656 (74.1)
BMI, kg/m^2 (mean, SD)	27.5 ± 4.2	27.3 ± 4.2	28.2 ± 4.5
Smoking habit			
No	632 (56.1)	342 (47.8)	347 (39.2)
Current smoker or stopped < 6 months	247 (22.0)	162 (22.6)	143 (16.2)
Past smoker	238 (21.2)	212 (29.6)	174 (19.7)
Unknown	7 (0.6)		221 (25.0)
Disease history			
Hypertension	936 (83.3)	564 (78.8)	725 (81.9)
Unknown			4 (0.5)
Diabetes	235 (20.9)	145 (20.3)	262 (29.6)
Unknown			1 (0.1)
Myocardial infarction	349 (31.0)	306 (42.7)	196 (22.1)
Unknown			197 (22.3)
Co-medication at baseline			
Beta-blocker	900 (80.1)	559 (78.1)	636 (71.9)
Unknown			43 (4.9)
ACE inhibitor	422 (37.5)	267 (37.3)	337 (38.1)
Unknown		1 (0.1)	270 (30.5)
Calcium channel blocker	314 (27.9)	260 (36.3)	190 (21.5)
Unknown			44 (5.0)
ARB	204 (18.1)	102 (14.2)	106 (12.0)
Unknown			369 (30.4)
Diuretic	313 (27.8)	125 (17.5)	NA
Statin or fibrate	1017 (90.5)	580 (81.0)	630 (71.2)
Unknown			29 (3.3)
Proton pump inhibitor	454 (40.4)	195 (27.2)	286 (32.3)
Unknown			22 (2.5)
Impaired renal function	9 (0.8)	4 (0.6)	19 (2.1)
Unknown		122 (17.0)	112 (12.7)
Clopidogrel loading	395 (35.1)	370 (51.7)	NA
Unknown		12 (1.7)	
CYP2C19 metabolizer			
Normal	821 (73.0)	505 (70.5)	620 (70.1)
Intermediate	274 (24.4)	183 (25.6)	241 (27.2)
Poor	29 (2.6)	20 (2.8)	18 (2.0)
Unknown	-	8 (1.1)	6 (0.7)
*CYP3A4*22*			
MAF	6.3%		
*1/*1	987 (87.8)	NA	NA
*1/*22	132 (11.7)		

*22/*22	5 (0.4)		
PPAR-α G209A			
MAF	25.4%	25.0%	27.2%
GG	630 (56.0)	402 (56.1)	462 (52.2)
GA	417 (37.1)	270 (37.7)	364 (41.1)
AA	77 (6.9)	44 (6.1)	59 (6.7)
PPAR-α A208G			
MAF	27.0%	26.3%	28.8%
AA	597 (53.1)	387 (54.1)	442 (49.9)
AG	448 (39.9)	282 (39.4)	377 (42.6)
GG	79 (7.0)	47 (6.6)	66 (7.5)

Note:
*CYP3A4*22* = rs35599367, G209A = rs4253728, A208G = rs4823613
Abbreviations: ACE = angiotensin converting enzyme, ARB = angiotensin receptor blocker, BMI = body mass index, MAF = minor allele frequency, NA = not available, PCI = percutaneous coronary intervention, SD = standard deviation.

Online Supplemental Table S3.
Estimates for the on-treatment platelet reactivity outcome in carriers of recessive alleles of *PPAR-α* G209A, and *PPAR-α* A208G in the POPular validation cohort

Genetic variant	Mean ± SD	Coefficient (95% CI)	
		**Crude**	**Adjusted**
**All patients (n = 716)**			
PPAR-α G209A			
GG or GA (n = 672)	213.0 ± 76.5	Ref	Ref
AA (n = 44)	202.5 ± 71.3	-10.54 (-33.81, 12.73)	-11.31 (-34.52, 11.89)ǂ
PPAR-α A208G			
AA or AG (n = 669)	212.8 ± 76.6	Ref	Ref
GG (n = 47)	205.9 ± 69.2	-7.0 (-29.52, 15.62)	-7.40 (-29.77, 14.98)ǂ
**In statin/fibrate users (n = 567)**			
PPAR-α G209A			
GG or GA (n = 533)	211.5 ± 77.2	Ref	Ref
AA (n = 34)	192.7 ± 64.3	-18.06 (-44.62, 8.49)	-15.28 (-42.15, 11.60)[§]
PPAR-α A208G			
AA or AG (n = 529)	211.8 ± 77.3	Ref	Ref
GG (n = 38)	197.2 ± 63.1	-14.54 (-39.76, 10.68)	-13.06 (-38.28, 12.15)[§]
**In statin/fibrate non-users (n = 146)**			
PPAR-α G209A			
GG or GA (n = 136)	218.3 ± 72.0	Ref	Ref
AA (n = 10)	231.9 ± 86.9	13.59 (-33.70, 60.89)	11.83 (-37.30, 60.95)[§]
PPAR-α A208G			
AA or AG (n = 137)	217.7 ± 72.1	Ref	Ref
GG (n = 9)	242.3 ± 85.3	24.61 (-24.95, 74.17)	27.93 (-22.83, 78.69)[§]

Note: All values are in PRU.
*CYP3A4*22* = rs35599367, G209A = rs4253728, A208G = rs4823613
Abbreviations: CI = confidence interval, SD = standard deviation, Ref = reference. All p-values for crude and adjusted odds ratios are > 0.05.

Symbols:
ǂ Adjusted for age, sex, body mass index, smoking, diabetes, previous myocardial infarction, co-medication (calcium channel blockers, statins/fibrates, proton pump inhibitors), impaired renal function, clopidogrel loading, and CYP2C19 metabolizer status.
§ Adjusted for all variables mentioned above, excluding statin/fibrate use

Online Supplemental Table S4. Odds ratio for high platelet reactivity outcome in carriers of recessive alleles of *PPAR-α* G209A, and *PPAR-α* A208G in the POPular validation cohort

PRU >= 236 PRU cut off

**Genetic variant**	**HPR+ (n = 290)**	**HPR- (n = 439)**	**Crude OR (95% CI)**	**Adjusted OR (95% CI)**
**All patients (n = 729)**				
PPAR-α G209A				
GG or GA (n = 685)	277 (40.4)	408 (59.6)	Ref	Ref
AA (n = 44)	13 (29.5)	31 (70.5)	0.62 (0.62, 1.20)	0.58 (0.27, 1.23)*
PPAR-α A208G				
AA or AG (n = 681)	275 (40.4)	406 (59.6)	Ref	Ref
GG (n = 48)	15 (31.3)	33 (68.8)	0.67 (0.36, 1.26)	0.64 (0.31, 1.30)*
**In statin/fibrate users (n = 579)**				
PPAR-α G209A				
GG or GA (n = 545)	220 (40.4)	325 (59.6)	Ref	Ref
AA (n = 34)	9 (26.5)	25 (73.5)	0.53 (0.24, 1.16)	0.54 (0.22, 1.33)[§]
PPAR-α A208G				
AA or AG (n = 540)	218 (40.4)	322 (59.6)	Ref	Ref
GG (n = 39)	11 (28.2)	28 (71.8)	0.58 (0.28, 1.19)	0.58 (0.26, 1.31)[§]
**In statin/fibrate non-users (n = 147)**				
PPAR-α G209A				
GG or GA (n = 137)	56 (40.9)	81 (59.1)	Ref	Ref
AA (n = 10)	4 (40.0)	6 (60.0)	0.96 (0.26, 3.58)	0.81 (0.16, 4.13)[§]
PPAR-α A208G				
GG or GA (n = 138)	56 (40.6)	82 (59.4)	Ref	Ref
AA (n = 9)	4 (44.4)	5 (55.6)	1.17 (0.30, 4.56)	1.27 (0.24, 6.72)[§]

PRU > 208 PRU cut off

**Genetic variant**	**HPR+ (n = 436)**	**HPR- (n = 688)**	**Crude OR (95% CI)**	**Adjusted OR (95% CI)**
**All patients (n = 729)**				
PPAR-α G209A				
GG or GA (n = 685)	365 (53.3)	320 (46.7)	Ref	Ref
AA (n = 44)	19 (43.2)	25 (56.8)	0.67 (0.36, 1.23)	0.76 (0.38, 1.53)*
PPAR-α A208G				
AA or AG (n = 681)	362 (53.2)	319 (46.8)	Ref	Ref
GG (n = 48)	22 (45.8)	26 (54.2)	0.75 (0.41, 1.34)	0.84 (0.43, 1.64)*
**In statin/fibrate users (n = 579)**				
PPAR-α G209A				
GG or GA (n = 545)	286 (52.5)	259 (47.5)	Ref	Ref
AA (n = 34)	13 (38.2)	21 (61.8)	0.56 (0.28, 1.14)	0.69 (0.30, 1.56)[§]
PPAR-α A208G				
AA or AG (n = 540)	283 (52.4)	257 (47.6)	Ref	Ref
GG (n = 39)	16 (41.0)	23 (59.0)	0.63 (0.33, 1.22)	0.73 (0.35, 1.56)[§]
**In statin/fibrate non-users (n = 147)**				
PPAR-α G209A				
GG or GA (n = 137)	78 (56.9)	59 (43.1)	Ref	Ref
AA (n = 10)	6 (60.0)	4 (40.0)	1.14 (0.31, 4.20)	1.32 (0.28, 6.34)[§]
PPAR-α A208G				
GG or GA (n = 138)	78 (56.5)	60 (43.5)	Ref	Ref
AA (n = 9)	6 (66.7)	3 (33.3)	1.54 (0.37, 6.40)	2.27 (0.43, 11.93)[§]

Note:
*CYP3A4*22* = rs35599367, G209A = rs4253728, A208G = rs4823613
Abbreviations: CI = confidence interval, HPR = high on-treatment platelet reactivity, OR = odds ratio, Ref = reference. All p-values for crude and adjusted odds ratios are > 0.05.
* Adjusted for age, sex, body mass index, smoking, diabetes, previous myocardial infarction, co-medication (calcium channel blockers, statins/fibrates, proton pump inhibitors), impaired renal function, clopidogrel loading, and CYP2C19 metabolizer status.
§ Adjusted for all variables mentioned above, excluding statin/fibrate use

Online Supplemental Table S5. Estimates for the on-treatment platelet reactivity outcome in carriers of recessive alleles of *PPAR-α* G209A, and *PPAR-α* A208G in the ICPC validation cohort

Genetic variant	Beta (95% CI)	Coefficient
		Crude p-value
**All patients (n = 882)**		
PPAR-α G209A		
GG or GA (n = 823)		Ref
AA (n = 59)	-0.028 (-0.024, 0.010)	p = 0.40
PPAR-α A208G		
AA or AG (n = 816)		Ref
GG (n = 66)	-0.030 (-0.025, 0.010)	p = 0.46
**In statin/fibrate users (n = 628)**		
PPAR-α G209A		
GG or GA (n = 592)		Ref
AA (n = 36)	-0.021 (-0.024, 0.013)	p = 0.59
PPAR-α A208G		
AA or AG (n = 587)		Ref
GG (n = 41)	-0.029 (-0.027, 0.012)	p = 0.46
**In statin/fibrate non-users (n = 225)**		
PPAR-α G209A		
GG or GA (n = 204)		Ref
AA (n = 21)	-0.050 (-0.052, 0.023)	p = 0.45
PPAR-α A208G		
AA or AG n = 202)		Ref
GG (n = 23)	-0.038 (-0.050, 0.028)	p = 0.57

Note:
*CYP3A4*22* = rs35599367, G209A = rs4253728, A208G = rs4823613
Abbreviations: CI = confidence interval, SD = standard deviation, Ref = reference.

Online Supplemental Figure S1:

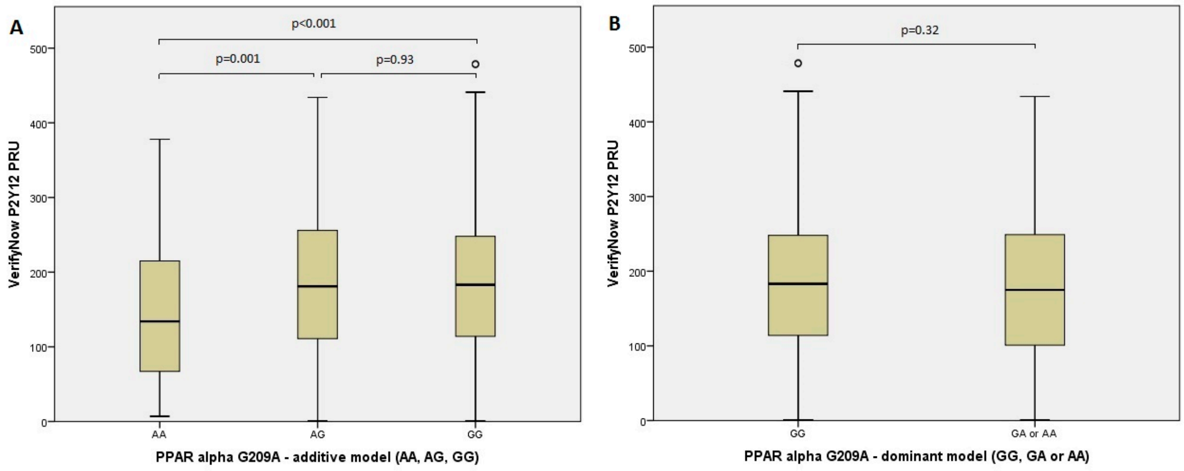

Online Supplemental Figure S1. The on-treatment platelet reactivity in the main analysis cohort, as measured with the VerifyNow® assay for PPAR alpha G209A (rs4253728), analyzed in a (a) additive model, (b) dominant model.

Online Supplemental Figure S2:

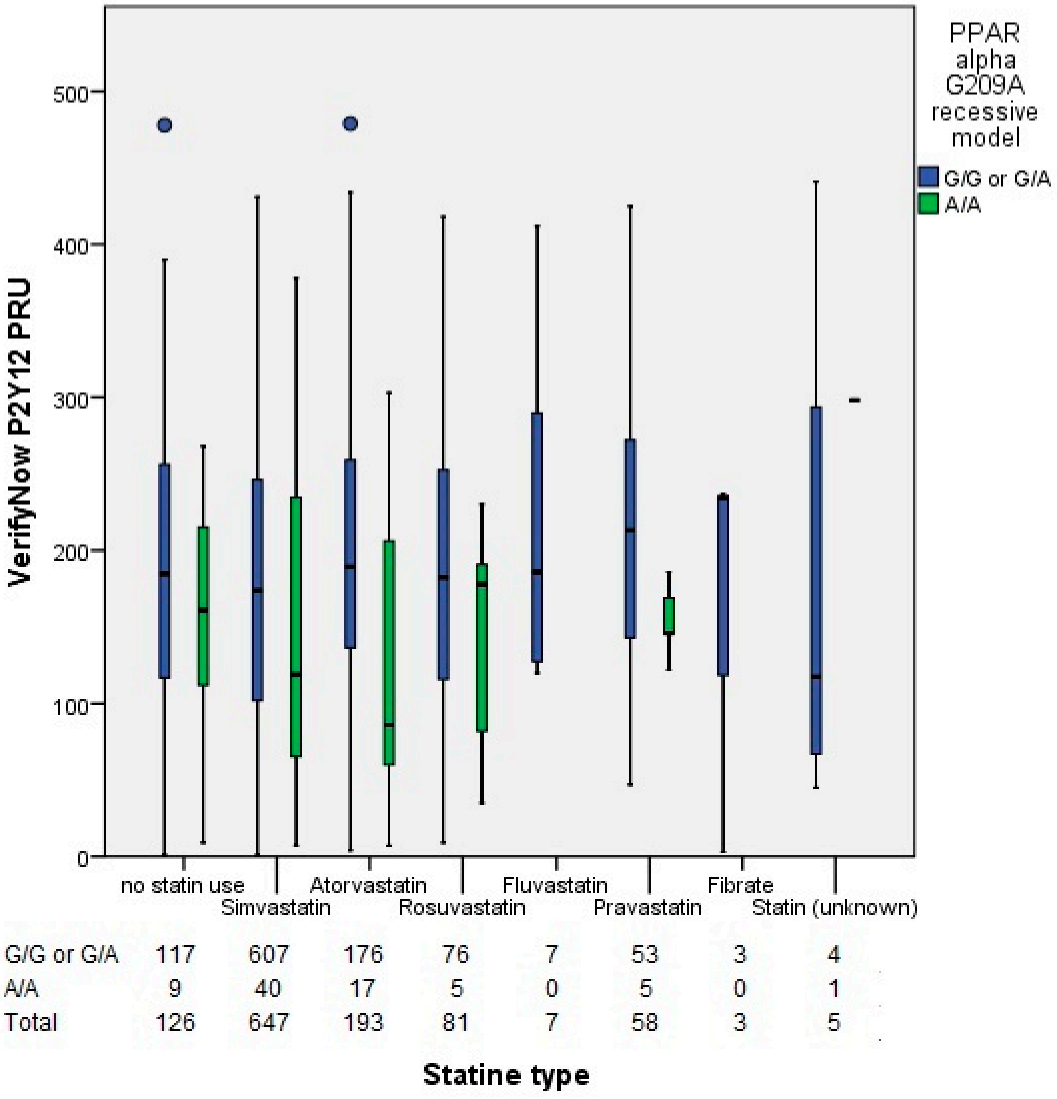

Online Supplemental Figure S2. The on-treatment platelet reactivity in the main analysis cohort, as measured with the VerifyNow® assay for PPAR alpha G209A (rs4253728), stratified according to different statin and fibrate use

Online Supplemental Figure S3:

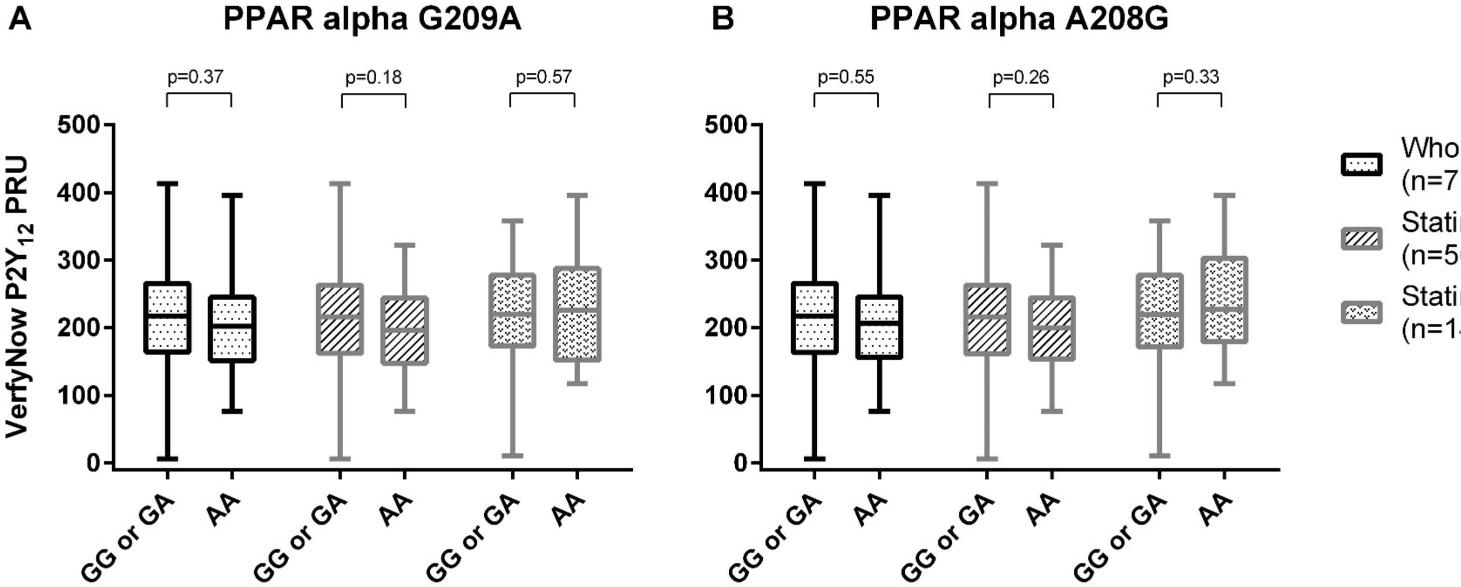

Online Supplemental Figure S3. The on-treatment platelet reactivity, as measured with the VerifyNow® assay: (a) *PPAR-α* G209A (rs4253728), (b) *PPAR-α* A208G (rs4823613) in the POPular validation cohort.

Online Supplementary Data:

International Clopidogrel Pharmacogenomic Consortium (ICPC) author list:

Thomas O. Bergmeijer, MD[1], Jean-Luc Reny, MD[2], Ruth E. Pakyz, MS[3], Li Gong, PhD[4], Joshua P. Lewis, PhD[3], Eun-Young Kim, MD[5], Daniel Aradi, MD[6], Israel Fernandez-Cadenas, PhD[7], Richard B. Horenstein, MD[3], Joshua D. Backman, PhD,[3] Ming Ta Michael Lee, PhD[8], Ryan M. Whaley[4], Joan Montaner, MD[9], Gian Franco Gensini, MD[10], John H. Cleator, MD[11], Kiyuk Chang, MD[12], Lene Holmvang, MD[13], Willibald Hochholzer, MD[14], Dan M. Roden, MD[15], Stefan Winter, PhD[16], Russ Altman, MD[4], Dimitrios Alexopoulos, MD[17], Ho-Sook Kim, PhD[18], Jean-Pierre Déry, MD[19], Meinrad Gawaz, MD[20], Kevin Bliden, MBA[21], Marco Valgimigli, MD[22], Rossella Marcucci, MD[23], Gianluca Campo, MD[24], Elke Schaeffeler, PhD[16], Nadia P. Dridi, MD[13], Ming-Shien Wen MD[34], Jae Gook Shin, MD[18], Tabassome Simon, MD[25], Pierre Fontana, MD[26], Betti Giusti, PhD[23], Tobias Geisler, MD[20], Michiaki Kubo, MD[27], Dietmar Trenk, PhD[28], Jolanta M. Siller-Matula, MD[29], Jurriën M. ten Berg, MD, PhD[1], Paul A. Gurbel, MD[21], Jean-Sebastien Hulot, MD[30], Braxton D. Mitchell, PhD[31], Matthias Schwab, MD[16,32], Shefali S. Verma, PhD[33], Yuki Bradford, MS[33], Marylyn D. Ritchie, PhD[33], Teri E. Klein, PhD[4], Alan R. Shuldiner, MD[3]

Affiliations:

[1]St Antonius Center for Platelet Function Research, Department of Cardiology, St Antonius Hospital Nieuwegein, The Netherlands
[2]Internal Medicine, Béziers Hospital, France; Geneva Platelet Group, University of Geneva School of Medicine; Department of Internal Medicine, Rehabilitation and Geriatrics, University Hospitals of Geneva, Switzerland
[3] Department of Medicine, Program for Personalized and Genomic Medicine, University of Maryland, Baltimore, USA
[4]Department of Biomedical Data Science, Stanford University, California, USA
[5]Department of Clinical Pharmacology, Inje University Busan Paik Hospital, South Korea
[6]Department of Cardiology, Heart Center Balatonfüred, Semmelweis University Budapest, Hungary
[7]Stroke Pharmacogenomics and Genetics, Fundació Docencia i Recerca Mutuaterrassa, Barcelona, Spain; Department of Neurology, Vall d'Hebron Institute of Research, Barcelona, Spain
[8]Genomic Medicine Institute, Geisinger Health System, Danville, PA, USA
[9]Neurovascular Research Laboratory, Vall d'Hebron Institute of Research, Barcelona, Spain
[10]Department of Experimental and Clinical Medicine, University of Florence, Florence, Italy

[11]Department of Cardiology and Department of Pharmacology, Vanderbilt University Medical Center, Nashville TN, USA
[12]Department of Internal Medicine, Cardiology Division, Seoul St. Mary's Hospital, The Catholic University of Korea, Seoul, South Korea
[13]Department of Cardiology, Rigshospitalet, Copenhagen University Hospital, Copenhagen, Denmark
[14]Department of Cardiology and Angiology II, University Heart Center Freiburg, Bad Krozingen, Germany
[15]Departments of Medicine, Pharmacology, and Biomedical Informatics, Vanderbilt University School of Medicine, Nashville TN, USA
[16]Dr Margarete Fischer-Bosch Institute of Clinical Pharmacology, Stuttgart, Germany; Department of Cardiology, University of Tuebingen, Tuebingen, Germany
[17]Department of Cardiology, Patras University Hospital, Patras, Greece
[18]Department of Clinical Pharmacology, Inje University, Busan Paik Hospital, Busan, South Korea
[19]Quebec Heart and Lung Institute, University Laval, Quebec City, Canada
[20]Department of Cardiology and Angiology, University of Teubingen, Teubingen, Germany
[21]Center for Thrombosis Research and Drug Development, Inova Heart and Vascular Institute, Falls Church, VA, USA
[22]Department of Cardiology, Bern University Hospital, Bern, Switzerland
[23]Department of Experimental and Clinical Medicine, University of Florence, Florence, Italy; Atherothrombotic Diseases Center, Careggi University Hospital, Florence, Italy
[24]Department of Cardiology, University Hospital of Ferrara, Ferrara, Italy; GVM Care & Rsearch Maria Cecilia Hospital, Cotignola, Italy
[25]APHP, Saint Antoine Hospital, Paris, France
[26] Department of Medicine, Geneva Platelet Group, University of Geneva School of Medicine, University Hospitals of Geneva, Switzerland; Division of Angiology and Haemostasis, University Hospitals of Geneva, Switzerland
[27]Laboratory for Genotyping Development, RIKEN Center for Integrative Medical Sciences, Yokohama, Japan
[28]Department of Cardiology and Angiology II, Clinical Pharmacology, University Heart Centre Freiburg, Bad Krozingen, Germany
[29]Department of Cardiology, Medical University of Vienna, Vienna, Austria
[30]Sorbonne Universités, UPMC Univ Paris 06, Institute of Cardiometabolism and Nutrition (ICAN), Pitié-Salpêtrière Hospital, F-75013 Paris, France
[31]Department of Medicine and Program for Personalized and Genomic Medicine, University of Maryland, Baltimore MD, USA; Geriatrics Research and Education Clinical Center, Baltimore Veterans Administration Medical Center, Baltimore MD, USA
[32]Departments of Cardiology, Clinical Pharmacology, Pharmacy and Biochemistry, University if Tuebingen, Tuebingen, Germany; Dr Margarete Fischer-Bosch Institute of Clinical Pharmacology, Stuttgart, Germany
[33]Center for Translational Bioinformatics, Institute for Biomedical Informatics, University of Pennsylvania, Philadelphia, PA, USA
[34]Division of Cardiology, Department of Internal Medicine, Chang Gung Memorial Hospital, Linkou and School of Medicine, Chang Gung University, Guishan Dist., Taoyuan City, Taiwan
